# Risk of atypical hyperplasia and endometrial carcinoma after initial diagnosis of non-atypical endometrial hyperplasia: A long-term follow-up study

**DOI:** 10.1371/journal.pone.0266339

**Published:** 2022-04-12

**Authors:** Clara M. Prip, Maria Stentebjerg, Mary H. Bennetsen, Lone K. Petersen, Pinar Bor

**Affiliations:** 1 Department of Obstetrics and Gynecology, Randers Regional Hospital, Randers, Denmark; 2 Department of Pathology, Randers Regional Hospital, Randers, Denmark; 3 Department of Obstetrics and Gynecology, Odense University Hospital, Odense, Denmark; 4 Open Patient Explorative Data Network, Odense University Hospital, Odense, Denmark; 5 Department of Clinical Research, University of Southern Denmark, Odense, Denmark; Dipartimento di Scienze Mediche e Chirugiche (DIMEC), Orsola Hospital, ITALY

## Abstract

**Objectives:**

The strong association between atypical endometrial hyperplasia and endometrial carcinoma is well established, but data on the risk of atypical hyperplasia and carcinoma in Danish women with non-atypical endometrial hyperplasia are almost non-existent. This study aimed to investigate the prevalence of atypical hyperplasia and endometrial carcinoma diagnosed within 3 months of initial diagnosis (defined as concurrent disease) and the risk of atypical hyperplasia and carcinoma more than 3 months after initial diagnosis (classified as progressive disease) in Danish women initially diagnosed with non-atypical endometrial hyperplasia.

**Design:**

This cohort study recruited 102 women diagnosed with non-atypical endometrial hyperplasia at Randers Regional Hospital in Randers, Denmark, between 2000 and 2015.

**Methods:**

The endometrium was evaluated by transvaginal ultrasound examination and office mini-hysteroscopy with biopsies in all non-hysterectomized women. Data regarding subsequent hysterectomy or endometrial sampling were obtained from medical records and the Danish Pathology Registry (Patobank).

**Results:**

A total of 15 women were diagnosed with atypical hyperplasia or carcinoma during follow-up. Concurrent atypical hyperplasia or carcinoma was seen in 2.9% (3/102), and among women who remained at risk for more than 3 months after initial diagnosis of non-atypical endometrial hyperplasia (*n* = 94), progression to atypical hyperplasia or carcinoma was seen in 13% (median follow-up 5.2 years, range 3.6 months to 15.1 years). Sixty-six percent of the women with progressive disease were diagnosed with atypical hyperplasia or carcinoma more than 1 year after initial diagnosis, but only two were diagnosed later than 5 years (5.2 and 9 years).

**Conclusions:**

The risk of being diagnosed with atypical endometrial hyperplasia or endometrial carcinoma more than 5 years after an initial diagnosis of non-atypical endometrial hyperplasia seems to be low in Danish women. Specialized follow-up more than 5 years after diagnosis of non-atypical endometrial hyperplasia may not be warranted.

## Introduction

Endometrial hyperplasia is considered a precursor of endometrial carcinoma, which is the sixth most commonly diagnosed cancer in women worldwide [1]. However, obtaining dependable estimates of the true incidence of endometrial hyperplasia is challenging for many reasons (e.g., changes in diagnostic criteria and methods, variation in hormone therapy, concomitant carcinoma, etc.).

According to the World Health Organization (WHO) classification from 2014, endometrial hyperplasia is classified as either *atypical hyperplasia* or *non-atypical hyperplasia* [2]. The distinction between atypical and non-atypical hyperplasia has been shown to be of great clinical importance because atypical hyperplasia is considered a premalignant condition [3–5]. Studies have reported concurrent endometrial carcinoma in 32–37% of cases, and a progression to carcinoma in 28% within 20 years despite hormonal treatment [3–5]. Consequently, women with atypical hyperplasia are most often treated in the same way as patients with endometrial carcinoma (i.e., with hysterectomy).

By contrast, there is no consensus on the management of women with non-atypical hyperplasia, and evidence-based, standardized guidelines for clinical follow-up are lacking. Several studies have investigated the risk of carcinoma after an initial diagnosis of non-atypical hyperplasia, but most of them are limited by their design (retrospective and single or two center studies) and small sample size (*n* = 15–354) [6–9]. Furthermore, several studies include data that makes it almost impossible to distinguish between occult carcinoma missed by the initial diagnostic test and progressive disease, because they have no defined time between initial hyperplasia diagnosis and carcinoma diagnosis [10]. A few studies have tried to distinguish between occult carcinoma and progressive disease by setting a cut-off at 1 year between hyperplasia and carcinoma diagnosis, and they report that <10% will progress to carcinoma within 20 years regardless of hormonal treatment [3, 11]. However, others question whether a 1-year interval is too long and argue that 3 months is a more appropriate cut-off [5].

Data on the risk of endometrial carcinoma in women initially diagnosed with non-atypical hyperplasia with an appropriate interval between initial hyperplasia diagnosis and carcinoma diagnosis is lacking and almost non-existent in Danish women. Therefore, the aim of this study was to estimate the prevalence of atypia or carcinoma diagnosed within 3 months of initial diagnosis (defined as concurrent disease) and the risk of atypia or carcinoma more than 3 months after initial diagnosis (classified as progressive disease) in Danish women initially diagnosed with non-atypical hyperplasia. Furthermore, we evaluated the need for long-term follow-up after an initial diagnosis of non-atypical hyperplasia.

## Materials and methods

### Study design and participants

This study is a cohort study of a retrospectively identified group of women diagnosed with non-atypical hyperplasia with a follow-up time of up to 15 years. Women eligible for inclusion were all those diagnosed with histologically verified non-atypical endometrial hyperplasia between January 1, 2000, and December 31, 2015 at Randers Regional Hospital in Randers, Denmark, who did not have a diagnosis of atypia or endometrial carcinoma in the initial sample or previous samples. They were identified using Patobank, a national database to which it is mandatory for all Danish pathology departments to report the results of patho-anatomical examinations [12, 13]. Patobank was searched for women using the SNOMED codes M72000 (hyperplasia) in combination with T84000 (endometrium) and/or T82000 (uterus) [14]. Women were excluded if the initial diagnosis of non-atypical hyperplasia was determined histologically in a hysterectomy specimen, if they could not understand Danish, or if no medical records could be obtained.

### Study setting and data collection

At follow-up, participant characteristics were obtained from medical records, and a structured interview conducted by the same interviewer. The non-hysterectomized women were invited to a face-to-face interview, a transvaginal ultrasound examination, and office mini-hysteroscopy with biopsies in the outpatient clinic at the Department of Obstetrics and Gynecology at Randers Regional Hospital, while the hysterectomized women were invited to participate in the project via a telephone interview. All examinations were conducted by the same gynecologist, who subspecialized in minimally invasive gynecological surgery. Information on the deceased women was obtained from medical records and from the results of patho-anatomical examinations stored in Patobank taken prior to their death.

A transvaginal ultrasound was performed using Voluson E6. Office mini-hysteroscopy was performed on all women, regardless of the transvaginal ultrasound findings. Local anesthesia was offered to women for whom the hysteroscopy procedure caused unacceptable discomfort. The entire cavity was visualized, and three 3–5 mm endometrial biopsies were taken from the fundus uteri and the posterior and anterior walls with a hysteroscopy biopsy cup (hereafter referred to as “follow-up biopsies”). The follow-up biopsies were examined by two gynecological histopathologists. Immunohistochemical staining for Ki-67 (ready-to-use, clone 30–9, Roche Diagnostics/Ventana Medical Systems, Inc., Tucson, AZ, USA) was performed whenever necessary to assess the proliferative activity. Persistence or progression of endometrial hyperplasia or endometrial carcinoma was defined using the WHO 1994 classifications, which divides endometrial hyperplasia into four types, depending on both the presence of cell atypia and glandular complexity, because this was the classification used when the initial diagnoses of non-atypical hyperplasia were made [15]: *simple non-atypical hyperplasia*, *complex non-atypical hyperplasia*, *simple atypical hyperplasia*, and *complex atypical hyperplasia*. However, in most of the analyses, the first two groups were grouped together as non-atypical hyperplasia, and the last two groups with atypia were grouped together as atypical hyperplasia according to the WHO 2014 classification [2].

### Data confidentiality and ethics

The study was conducted in accordance with the Declaration of Helsinki and approved by the Danish Data Protection Agency (J.no. 2017-41-5129) and the Danish National Committee on Health Research Ethics (Case no. 1-10-72-432-17). Oral and written consent for the use of data from interviews, medical records, clinical examinations, and biopsies for research purposes was obtained from women at time of inclusion. Data on the deceased women were obtained from medical records and Patobank after approval by the Danish Data Protection Agency and the Danish National Committee on Health Research Ethics.

### Statistical analyses

The data were managed using REDCap (Research Electronic Data Capture) [16]. Statistical analyses were performed using Stata version 16 software [17]. Numerical values were presented as medians with minimum and maximum values. Kaplan–Meier analysis was performed to evaluate the absence of atypical hyperplasia or carcinoma. The observation time for the hysterectomized women ended at the date of their hysterectomy, and the non-hysterectomized women were observed until their follow-up examinations or death.

## Results

**Fig 1:**
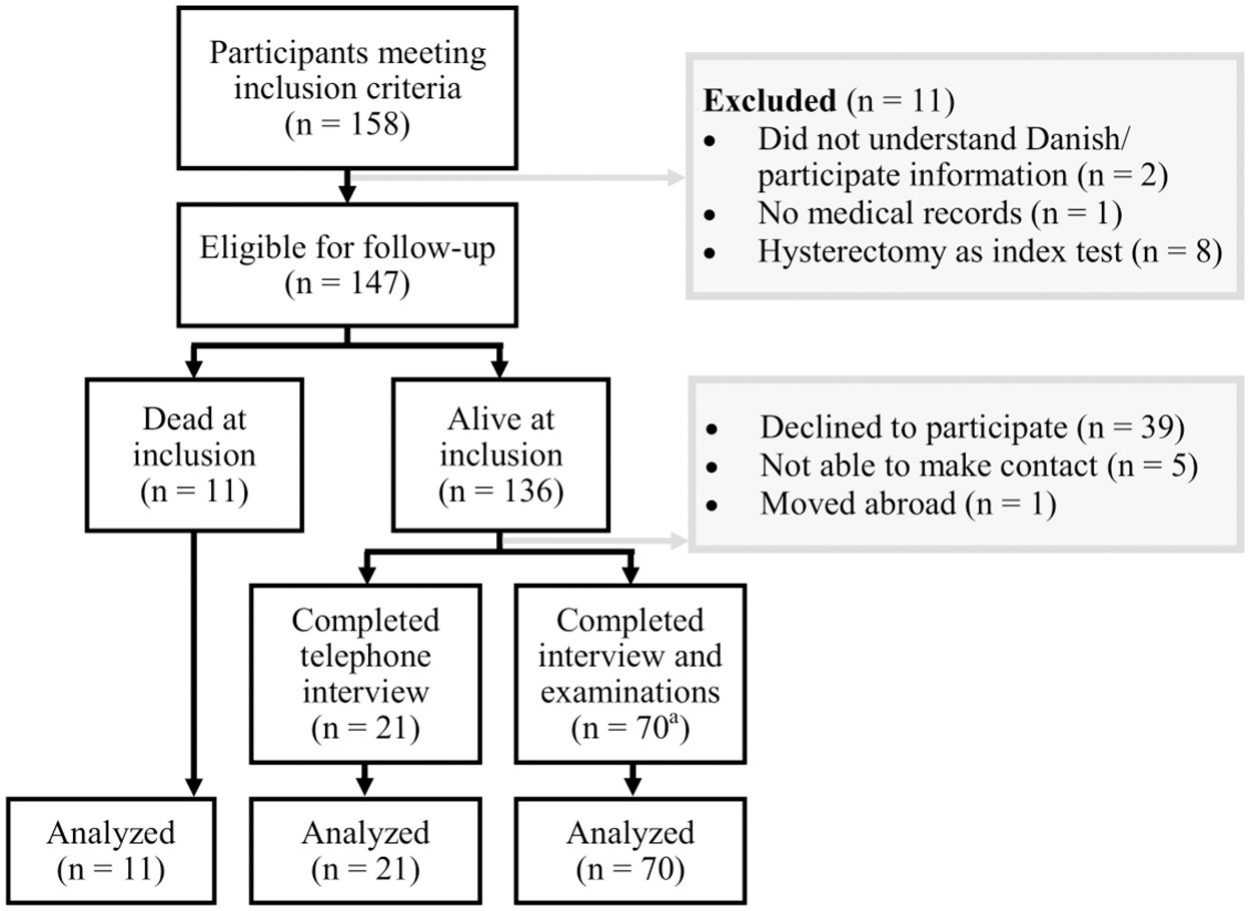
Participant flow chart. Index test = initial histological sample with non-atypical endometrial hyperplasia between 2000–2015. a) One woman did not want to participate in office mini-hysteroscopy, but she completed the transvaginal ultrasound and the interview.

The search in Patobank identified 158 women who met the inclusion criteria (Fig 1). Eleven women were excluded (Fig 1). Ninety-one women accepted participation in the study, gave their informed consent, and entered the final analyses. Of the 91 women, 21 had had a hysterectomy and completed only the telephone interview, while 70 women completed the clinical examinations and the interview. Biopsies sufficient for diagnostic evaluation were obtained in 55 women. Biopsy results are shown in Table 1. Some of the most common reasons for non-participation were comorbidity and not wanting to spend time taking part in the study. Age and results of patho-anatomical examinations of non-participants were recorded. Eleven women had died at the time of inclusion (Fig 1). These women were included in the final analyses based on the same criteria as the 91 women still alive. This meant that data which could not be obtained from medical records or Patobank in the deceased women were categorized as missing. There were only few missing data. Missing values in the deceased women included BMI at inclusion and endometrial thickness in three women along with information on pregnancy in one woman. Therefore, a total of 102 women were included the final analyses (Fig 1).

**Table 1:** Results of biopsies from office mini-hysteroscopy at follow-up.

	Non-atypical endometrial hyperplasia n = 69	
	n/total	%
Non-atypical hyperplasia	2/69	3
Atypical hyperplasia	1/69	1.5
Atrophy	28/69	40.5
Other^a^	24/69	35
Insufficient biopsies	5/69	7
No biopsies due to cervical stenosis	9/69	13

^a^) Other biopsy results include irregular proliferation (*n* = 3), proliferation phase (*n* = 1), normal tissue (*n* = 4), inactive endometrium (*n* = 1), and no sign of malignancy (*n* = 5).

The basic characteristics of the 102 women analyzed are shown in Table 2. The index test (histological sample indicating the initial diagnosis of non-atypical hyperplasia between 2000 and 2015) showed that 78 women (76.5%) had simple non-atypical hyperplasia, and 24 (23.5%) had complex non-atypical hyperplasia according to the WHO 1994 classification [15]. Thirty-three percent of them were postmenopausal at the time of the index test, and 94% had irregular bleeding or postmenopausal bleeding as indication for the index test (Table 2).

**Table 2:** Participant characteristics.

	Non-atypical endometrial hyperplasia n = 102	
	n/total	%
	---	---
	*median*	*min-max*
**Median age at index test (years)**	*52*.*5*, *n = 102*	*28–89*
**Median BMI at index test (kg/m** ^**2**^ **)** ^a^	*27*.*2*, *n = 91*	*17*.*7–59*.*5*
**Ever pregnant**	95/101	94
**Ever hypertension**	41/102	40
**Ever diabetes mellitus**	17/102	16.5
**Irregular or postmenopausal bleeding as indication for index test**	96/102	94
**Postmenopausal at index test**	40/102	39
**Index test**		
*Endometrial biopsy* ^b^	77/102	75.5
*Transcervical hysteroscopic endometrial resection*	22/102	21.5
*Hysteroscopy with biopsy*	3/102	3
**Endometrial thickness at index test in postmenopausal women**		
*<5 mm*	3/36	8.5
*5–10 mm*	15/36	41.5
*>10 mm*	18/36	50
**Endometrial histology in index test according to WHO 1994 classification** ^c^		
*Simple non-atypical hyperplasia*	78/102	76.5
*Complex non-atypical hyperplasia*	24/102	23.5

BMI = body mass index

WHO = World Health Organization

Index test = initial histological sample with non-atypical endometrial hyperplasia between 2000–2015

Numbers are presented as number out of total number with percentage. Unknowns are not counted as part of the total number in each group.

Medians are presented with minimum value–maximum value.

^a^) BMI of the women who had died at the time of inclusion are not included.

^b^) Endometrial sampling using Pipelle/Vabra.

^c^) Reference [15].

### Concurrent atypical hyperplasia or endometrial carcinoma

Three women were diagnosed with atypia or carcinoma within 3 months after the index test (Table 3). Two of them were diagnosed as part of a clinical follow-up program due to an index test with a histopathological suspicion of a more severe type of endometrial hyperplasia, and one woman had a transcervical hysteroscopic endometrial resection due to irregular bleeding showing atypia.

**Table 3:** Results on follow-up of women who had a hysterectomy within 3 months of initial diagnosis of non-atypical endometrial hyperplasia.

	n/total	%
**Hysterectomy within 3 months of index test**	8/8	100
*Indication for hysterectomy*:		
• Diagnosis of atypia or carcinoma	3/8	37.5
• Irregular or postmenopausal bleeding	1/8	12.5
• Other reason^a^	4/8	50

Index test = initial histological sample with non-atypical endometrial hyperplasia between 2000–2015

Numbers are presented as number out of total number with percentage.

^a^) Other reasons include ovarian tumor (*n* = 2), genital prolapse (*n* = 1), and patient desire (*n* = 1).

### Progression to atypical hyperplasia or endometrial carcinoma

**Fig 2:**
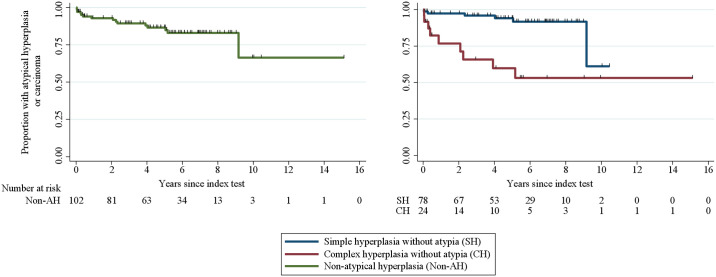
Subsequent atypical hyperplasia or endometrial carcinoma presented in all women with non-atypical hyperplasia (left) and according to WHO 1994 classification type of non-atypical hyperplasia (right). The endpoint was atypia or carcinoma. Women were censored (black lines) due to hysterectomy, follow-up biopsy, or death. The women in whom follow-up biopsies were not obtained or in whom the biopsy material obtained was insufficient for diagnostic evaluation were censored on the day of their follow-up clinical examination.

Ninety-four of the 102 women analyzed remained at risk more than 3 months after the index test; that is, they were not hysterectomized or diagnosed with atypia or carcinoma within 3 months after the index test (Table 4). They were followed up for a median of 5.2 years (3.6 months to 15.1 years), during which time 12 women (13%) were diagnosed with atypia or carcinoma (Table 4). Four women (4.5%) were diagnosed during a hospital-based follow-up program within 1 year after the index test. The remaining eight women (8.5%) were diagnosed with atypia or carcinoma 2 (*n* = 3), 4 (*n* = 2), 5 (*n* = 2), and 9 years after the index test (Table 4 and Fig 2). Six of them did not receive treatment or were under observation initially, one initially had a transcervical hysteroscopic endometrial resection, and one was primarily treated with oral progestin and observed until regression. They all had irregular bleeding between the index test and diagnosis of atypia or carcinoma.

**Table 4:** Results of follow-up in women who remained at risk more than three months after initial diagnosis of non-atypical endometrial hyperplasia.

	Non-atypical endometrial hyperplasia n = 94	
	n/total	%
	---	---
	*median*	*min-max*
**Median follow-up time (years)**	*5*.*2*, *n = 94*	*0*.*3–15*.*1*
**Diagnosis of atypia or carcinoma**		
*3–12 months after index test (years)*	4/94	4.5
*>12 months after index test (years)*	8/94	8.5
**Median time to progression to atypia or carcinoma (years)**	*2*.*3*, *n = 12*	*0*.*3–9*.*2*
**Intervention during follow-up period**		
*No intervention*	24/94	25.5
*Hormones only*	19/94	20.5
*Transcervical hysteroscopic endometrial resection* ^a^	33/94	35
*Hysterectomy* ^b^	18/94	19
Indication for hysterectomy:		
• Diagnosis of atypia or carcinoma	10/94	10.5
• Irregular or postmenopausal bleeding	5/94	5.5
• Other reason^c^	3/94	3

Index test = initial histological sample with non-atypical hyperplasia between 2000–2015

Numbers are presented as number out of total number with percentage.

Medians are presented with minimum value–maximum value.

^a^) Women who received both transcervical hysteroscopic endometrial resection and hormonal treatment are listed as having received transcervical hysteroscopic endometrial resection.

^b^) Women who had hormonal treatment or transcervical hysteroscopic endometrial resection prior to their hysterectomy are listed as having undergone hysterectomy.

^c^) Other reasons include uterine irregularity on a scan for discus prolapse (n = 1) and patient desire (n = 2).

### The hysterectomized women

A total of 26 women were hysterectomized. Eight women (30%) were hysterectomized within 3 months of the index test (Table 3). Three of them had a diagnosis of atypia or carcinoma in a retest done within 3 months of the index test. The remaining five women were hysterectomized due to irregular bleeding (*n* = 1), an ovarian tumor (*n* = 2), genital prolapse (*n* = 1), and patient desire (*n* = 1) (Table 3). The definitive histological diagnoses of these five were all benign (non-atypical hyperplasia: 1, no endometrial hyperplasia: 4).

Nine women (35%) were hysterectomized more than 3 months after the index test while they were followed in a hospital-based follow-up program, most of them within 1 year of the index test. Three of the nine women had a preoperative diagnosis of atypia or carcinoma from a retest done during clinical follow-up. The definitive histological diagnoses of the six women undergoing hysterectomy based on a preoperative diagnosis of non-atypical hyperplasia were all benign (non-atypical hyperplasia: 3, no endometrial hyperplasia: 3).

Nine women (35%) were hysterectomized more than 3 months after the index test after being referred to the hospital anew, most of them more than two years after the index test. Seven of them had a preoperative diagnosis of atypia or carcinoma. The definitive histological diagnoses of the two women undergoing hysterectomy for a non-oncologic indication (i.e., irregular bleeding and fibroma on a scan) were benign (no endometrial hyperplasia: 2).

### Definitive endometrial histology of the deceased women

Eleven women were dead at the time of inclusion (Fig 1). Seven women had a definitive endometrial histological diagnosis obtained from Patobank. Five of the seven were diagnosed with atypia or carcinoma, and two women had an autopsy with no sign of malignancy. Four women did not have a definitive endometrial histological diagnosis in Patobank; thus, their endometrial status at follow-up were unknown. However, it is mandatory for all Danish pathology departments to report to Patobank which therefore contains information on nearly 100% of all histological samples obtained in Danish patients. Therefore, it is unlikely that these 4 women have been diagnosed with endometrial pathology which has not been registered in Patobank.

## Discussion

In this cohort study of women initially diagnosed with non-atypical endometrial hyperplasia, 2.9% (3/102) of women were diagnosed with atypia or endometrial carcinoma within three months of the initial diagnosis of non-atypical hyperplasia. Among women who remained at risk more than 3 months after initial diagnosis (*n* = 94), progression (defined as diagnosis of atypia or carcinoma more than three months after initial diagnosis) was seen in 13% (median follow-up 5.2 years, range 3.6 months to 15.1 years). Sixty-six percent of the women with progressive disease were diagnosed with atypia or carcinoma more than 1 year after initial diagnosis, but only two were diagnosed later than 5 years (5.2 and 9 years).

Consistent with our results, previous studies found that 0–9% of a cohort of mixed pre- and postmenopausal women with non-atypical hyperplasia had endometrial carcinoma in a hysterectomy performed within 4 months [18, 19]. However, studies investigating concurrent carcinoma defined by a cut-off of 3 months or less, only including women diagnosed with non-atypical hyperplasia, are lacking [5].

As previously stated, there is a lack of studies on the risk of progression to endometrial carcinoma after a diagnosis of non-atypical hyperplasia, including women who were at risk for at least 3 months after initial diagnosis. However, the findings of the few existing studies are in line with the results of our study [3, 5, 11, 20]. A recent meta-analysis by Doherty et al. included studies published between 1994 and 2018 on the risk of endometrial carcinoma after an initial diagnosis of non-atypical hyperplasia and excluded studies with no defined time between hyperplasia and carcinoma diagnosis [5]. After exclusion of studies where rates per person-years could not be calculated (i.e., if they did not report mean or median follow-up time), Doherty et al. found only one study by Garuti et al. that investigated progression to carcinoma (defined as carcinoma detected more than 3 months after hyperplasia diagnosis) solely including non-atypical hyperplasia [21]. Garuti et al. reported progression in 12% (3/24) of women within 2 years of follow-up [21]. However, the study was limited by its low sample size and simultaneous Tamoxifen treatment. Two often-cited studies by Lacey et al. and Kurman et al., which reported on women with non-atypical hyperplasia who remained at risk for at least 1 year after initial diagnosis, were not included in the meta-analysis by Doherty et al. due to case-control design and year of origin (1985), respectively [3, 11]. They found a <10% progression to carcinoma within 20 years, regardless of hormonal treatment. To our knowledge, only one other study on Danish women with non-atypical hyperplasia exists [20]. It included 114 Danish women with complex non-atypical hyperplasia (according to the WHO 1994 classification [15]), regardless of the time between initial hyperplasia diagnosis and diagnosis of atypia or carcinoma. However, the authors reported separate data on women who remained at risk for at least 1 year after initial diagnosis and found that 9.4% (8/85) of the remaining women at risk developed atypia or carcinoma [20]. They concluded that hospital-based follow-up and any necessary treatment during the first 3–5 years after diagnosis could be warranted [20].

The present increase in the incidence of obesity worldwide carries the risk of more women being diagnosed with endometrial hyperplasia or carcinoma [22], but there is still no consensus on the management of women with non-atypical hyperplasia. A large study investigating the need for treatment and follow-up in women with non-atypical hyperplasia is still lacking.

In our study, we have tried to distinguish between concurrent carcinoma and progressive disease by setting a time limit between initial hyperplasia diagnosis and diagnosis of carcinoma, which is lacking in previous studies [5]. However, the recurring problem in distinguishing occult malignancy from progressive disease relates to the well-established challenges regarding diagnostic accuracy and the validity of the pathological evaluation. It is well known that the diagnostic accuracy of blind endometrial sampling, visually guided biopsy, and transcervical hysteroscopic endometrial resection differs, and that none of them have diagnostic accuracy as the gold standard hysterectomy [23–27]. Our study, along with the above-cited studies, may have misclassified the type of hyperplasia or missed concurrent carcinoma at inclusion due to the use of different diagnostic procedures with varying diagnostic precision as initial sampling methods [11, 28]. Moreover, in our study, the index tests were not reviewed, and the challenges regarding standardization in endometrial diagnostics are well established [29]. However, all index tests were performed by specialized gynecological histopathologists.

Our small sample size limited our progression analyses, and even though all non-participants could be accounted for, little is known about them, except their ages and the information stored in Patobank. However, the collected data from the non-participants were not significantly different from the participant data. The median age at index test among non-participants was 52 (23–86) years with a p-value of 0.4 (using the Kruskal–Wallis test) when compared with the median age of the participants. Two women (5%) out of the forty-two non-participants who remained at risk more than 3 months after the index test were diagnosed with atypia or carcinoma within the follow-up period; one within one year of the index test and the other 11.3 years after the index test. We cannot exclude possible selection bias in the recruitment of patients for this study. However, it seems unlikely that a woman with symptoms would decline participation. Thus, we estimate that the risk of selection bias is low.

We included women regardless of the treatment (hormonal or transcervical hysteroscopic endometrial resection) they were receiving, which could decrease the risk of atypia or carcinoma. Future studies should include a larger number of women to allow for stratification for treatment methods.

This study is the first Danish follow-up study to include women with all types of non-atypical hyperplasia with the intention of investigating the long-term risk of atypia or carcinoma. It is based on data from Patobank, which contains all pathology examinations conducted in Denmark. The data are registered in a uniform way, and there is an extremely low proportion of missing data [13]. We also consider it a great strength of the study that the histological samples from our participants at follow-up were all taken using the same method (i.e., office mini-hysteroscopy, which has been shown to have a higher diagnostic accuracy than blind sampling) [25, 27]. In addition, we attempted to distinguish between concurrent disease and progression to atypia or carcinoma by setting a cut-off at 3 months after their diagnosis of non-atypical hyperplasia and evaluated progression after 1 year. Moreover, we investigated the risk of both atypia and carcinoma, which we consider a strength because their strong association indicates that similar follow-up measures should be undertaken after their diagnosis.

The new WHO 2014 classification does not distinguish between simple and complex hyperplasia, unlike the 1994 classification [2, 15]. The differentiation between simple and complex was abandoned partly due to a high degree of interobserver variability between pathologists [30]. However, our data suggest that there is a considerable difference in the risk of being diagnosed with atypia or endometrial carcinoma during the follow-up period between women initially diagnosed with simple non-atypical hyperplasia and those diagnosed with complex non-atypical hyperplasia, with a higher risk after a diagnosis with complex non-atypical hyperplasia. Women with complex non-atypical hyperplasia who are shown to have occult or progressive disease are now grouped together with women with simple non-atypical hyperplasia. Consequently, they might not be diagnosed with atypia or carcinoma on time for sufficient treatment unless they make contact with the health system themselves, for example, if they have symptoms. Therefore, we suggest that future risk stratification and the resulting decision on need of clinical follow-up should be more individualized and include different parameters associated with risk of atypia or carcinoma, such as BMI [31], persisting symptoms (i.e., irregular bleeding) [32], and whether complex hyperplasia (i.e., a higher gland-to-stroma ratio) is present in the histological specimen. It can also be argued that future research in this field will instead change the focus from the need for future follow-up in women with non-atypical hyperplasia and put the focus on diagnostic precision, morbidity, and cost benefits of various diagnostic methods along with the need for individual counseling in a clinical follow-up setting, which will allow for qualified recommendations for follow-up.

## Conclusions

The risk of being diagnosed with atypia or carcinoma more than 5 years after initial diagnosis of non-atypical hyperplasia seems to be low in Danish women. Specialized follow-up more than 5 years after diagnosis of non-atypical hyperplasia may not be necessary. However, more studies on the long-term risk of progression are needed.

## Supporting information

S1 Dataset Values behind the median age at index test and median BMI at index test presented in Table 2. Index test = initial histological sample with non-atypical hyperplasia between 2000–2015. NA = Not available.
(XLSX)

S2 Dataset Values behind the graphs presented in Fig 2. Failure value 1 indicates a diagnosis with the endpoint (atypia or carcinoma). Failure value 0 indicates that the endpoint was not met, i.e., no diagnosis with atypia or carcinoma.
(XLSX)

S3 Dataset Values behind the median follow-up time and median time to progression to atypia or carcinoma presented in Table 4. Index test = initial histological sample with non-atypical hyperplasia between 2000–2015. NA = Not available.
(XLSX)
